# Application of Poloxamer for In Situ Eye Drop Modeling by Enrichment with Propolis and Balsam Poplar Buds Phenolic Compounds

**DOI:** 10.3390/gels10030161

**Published:** 2024-02-21

**Authors:** Monika Jokubaite, Mindaugas Marksa, Kristina Ramanauskiene

**Affiliations:** 1 Department of Drug Chemistry, Faculty of Pharmacy, Lithuanian University of Health Sciences, Sukileliai Avenue 13, LT-50162 Kaunas, Lithuania; 2 Department of Analytical & Toxicological Chemistry, Faculty of Pharmacy, Lithuanian University of Health Sciences, Sukileliai Avenue 13, LT-50162 Kaunas, Lithuania; mindaugas.marksa@lsmu.lt; 3 Department of Clinical Pharmacy, Faculty of Pharmacy, Lithuanian University of Health Sciences, Sukileliai Avenue 13, LT-50162 Kaunas, Lithuania; kristina.ramanauskiene@lsmu.lt

**Keywords:** gels, in situ, eye drops, propolis, balsam poplar, *p*-coumaric acid

## Abstract

In situ poloxamer-based gels are increasingly being explored as ocular drug delivery carriers to extend the release of active substances, thereby enhancing bioavailability. The objective of this study was to develop thermally stable in situ gels incorporating balsam poplar bud extract, propolis extract, and *p*-coumaric acid solution and to evaluate the physicochemical parameters of these gelified eye drops. This research assessed the compatibility of poloxamer-based eye drops with active components, their physicochemical properties, stability post-sterilization and during storage, and the release profiles of the active compounds. Fifteen eye drop formulations were prepared and categorized into three groups based on active components. One of the active components was propolis extract. As an alternative to propolis, eye drops containing the plant precursor, balsam poplar bud extract, were developed. The third group’s active component was *p*-coumaric acid, a dominant phenolic acid in propolis and balsam poplar bud extracts. The study reported phenolic contents of 76.63 CAE mg/g for propolis and 83.25 CAE mg/g for balsam poplar bud aqueous extracts, with balsam poplar bud extracts showing higher SPF values (14.0) compared to propolis (12.7), while *p*-coumaric acid solution exhibited the highest SPF values (25.5). All eye drops were transparent, with pH values meeting the requirements for ocular drops. Formulations containing 8–10% poloxamer 407 met the criteria for in situ gels. All formulations remained stable for 90 days. Conclusion: The study results indicate that the formulated gels possess suitable physicochemical properties, are resistant to applied autoclaving conditions, and exhibit an extended release of active compounds with an increase in poloxamer content.

## 1. Introduction

Dry eye syndrome, a prevalent ophthalmologic condition, significantly impacts patients’ quality of life by causing discomfort and visual disturbances due to inadequate tear fluid production or distribution [1]. This syndrome is especially common among adults and is associated with various symptoms that can severely affect day-to-day activities and overall well-being. Given the growing prevalence of dry eye syndrome and its substantial impact on patients’ quality of life, there is a need for effective treatments [2]. Current research is increasingly focused on the development of new therapeutic options, including naturally derived active ingredients for eye drop formulations. These natural components are being investigated for their potential protective effects against the symptoms of dry eye syndrome, offering a promising avenue for enhancing treatment efficacy. Liquid eye drop formulations are among the most commonly used forms [3]. The efficacy of ocular formulations is determined not only by the active ingredients but also by the choice of pharmaceutical form, excipients, and their quantities. Locally applied ocular drugs face challenges in effectively reaching deeper tissues due to various biological barriers [4,5]. Liquid eye drop formulations often result in transient effects, as the active substance is rapidly removed from the ocular surface due to tear drainage, blinking, and other factors [6,7]. Overcoming these obstacles and enhancing biological availability is a growing area of research interest. While emulsions and ointments have been used to extend the action of active compounds in the eye, they present drawbacks such as irritation, discomfort, and blurred vision [8]. Recent years have seen research into polymer-based active compound delivery systems, including thermally responsive, pH-responsive, and ion-responsive formulations, particularly those using materials like poloxamers [9]. Gelified eye drops prolong the release of active compounds from the carrier, thus improving bioavailability [10]. Notably, in situ gel drops gelify under physiological conditions, ensuring comfortable application. In situ gels made from poloxamer-based formulations due to temperature changes transform into a hydrogel on the eye surface, offering a promising new drug form characterized by prolonged drug retention and favorable release properties [11,12,13]. Poloxamer compositions with additional gelling agents using natural active substances are the focus of this study, aiming to create extended-release gelified drops with optimal physicochemical properties. This research aligns with the growing scientific interest in eye drops containing naturally derived phenolic compounds, whose positive effects on the eyes have been scientifically proven. Lodovici et al. evaluated the protective effect of *p*-coumaric acid against UVB-induced cellular damage in rabbit cornea in vivo. Their findings demonstrated that *p*-coumaric acid protects ocular tissues and reduces harmful UVB radiation effects at low concentrations, presumably due to its free radical scavenging and antioxidative properties. The results are promising, and *p*-coumaric acid could be an effective means of protecting the eyes from free radical damage caused by UV lamps, UVB solar radiation, or welding torches [14]. Global researchers are examining *p*-coumaric acid in eye drop formulations for its protective effect. Increasing attention is paid to *p*-coumaric and other biologically active compounds from natural sources. One widely studied source of phenolic compounds is the bee product propolis, known for its antioxidative, antibacterial, antifungal, and anti-inflammatory properties [15,16,17]. The extraction of propolis is a multi-step process, and it is important to investigate plant precursors as alternatives to propolis [18]. Propolis collected in Lithuania belongs to the poplar type. Study results showed that the chemical composition and biological activity profile of Lithuanian propolis extracts are similar to those of studied balsam poplar bud (*Populus balsamifera* L.) extracts [19,20]. Investigations into the biological activity and chemical composition of balsam poplar buds revealed antioxidative, antimicrobial, and UV-protective effects [19,21]. Given that *p*-coumaric acid is predominant in propolis and poplar bud extracts, their application in eye drop formulations is pertinent. These raw materials differ in origin, and the physicochemical properties of their extracts could influence the quality and stability of eye drops. Active compounds in the chemical composition of propolis and balsam poplar buds and their biological properties are relevant for alleviating the symptoms of dry eye syndrome. It is crucial to evaluate and compare the physicochemical properties of eye drops with *p*-coumaric acid, propolis, and poplar bud extracts: pH, viscosity, gelation temperature, and stability. The aim of this study is to model thermally stable in situ gels with balsam poplar bud extract, propolis extract, and *p*-coumaric acid solution and to evaluate the physicochemical parameters and stability of these gelified eye drops in vitro.

## 2. Results

### 2.1. Chemical Composition of Propolis and Balsam Poplar Bud Extracts

#### 2.1.1. Total Phenolic Compounds and SPF Values of Aqueous Extracts and *p*-Coumaric Acid Solution

In the aqueous extracts of propolis and balsam poplar buds, the total phenolic content was determined to be 76.63 ± 5.39 CAE mg/g and 83.25 ± 3.88 CAE mg/g of dry raw material, respectively (Table 1). No statistically significant difference was observed in the total phenolic content between the propolis and balsam poplar bud aqueous extracts (*p* > 0.05). The SPF values obtained were 12.7 for propolis extracts, 14.0 for balsam poplar bud extracts, and 25.5 for the *p*-coumaric acid solution at a concentration of 50 µg/mL. The aqueous extracts of balsam poplar buds exhibited higher SPF values compared to those of the propolis extracts. The 50 µg/mL solution of *p*-coumaric acid demonstrated significantly higher SPF values compared to both the propolis and balsam poplar bud extracts.

#### 2.1.2. HPLC Analysis

Using HPLC analysis, active compounds were identified in the aqueous extracts of balsam poplar buds and propolis (Table 2). In the balsam poplar bud extracts, exclusive compounds such as salicin, chlorogenic acid, cinnamic acid, pinobanksin, galangin, and ferulic acid were detected, with the salicin concentration reaching 112.07 ± 4.99 µg/mL. In propolis, vanillin (105.60 ± 5.58 µg/mL), vanillic acid, and ferulic acid were found—251.78 ± 19.00 µg/mL. The same active compounds, namely apigenin, caffeic acid, *p*-coumaric acid, and pinocembrin, were identified in both the balsam poplar bud and propolis aqueous extracts. It was determined that caffeic acid was more concentrated in propolis extracts (42.22 ± 2.95 µg/mL) compared to balsam poplar bud extracts (26.42 ± 1.09 µg/mL). *p*-Coumaric acid was identified as a dominant compound in both poplar buds and propolis extracts, with no statistically significant difference in *p*-coumaric acid concentration between the extracts (*p* > 0.05).

### 2.2. Composition of Formulations

In situ eye drops were prepared by weight/weight (*w*/*w*). The formulations of the in situ eye drops comprised a fixed concentration of 0.75% sodium carboxymethylcellulose (NaCMC) and a 7.5% active compound concentration (balsam poplar bud extracts, propolis aqueous extracts, *p*-coumaric acid), with the addition of purified water to make up 100% volume (Table 3). The variable polymer in all compositions was Poloxamer 407 (P407) at concentrations of 8%, 10%, 11%, 12%, and 13%. The varying concentrations of Poloxamer 407 in the in situ gels formulations allowed for the comparison of the polymer concentration’s impact on the physicochemical properties of the in situ gels.

### 2.3. Physicochemical Parameters of Modeled In Situ Eye Drops

#### 2.3.1. Experimental Eye Drops Viscosity

**Figure 1:**
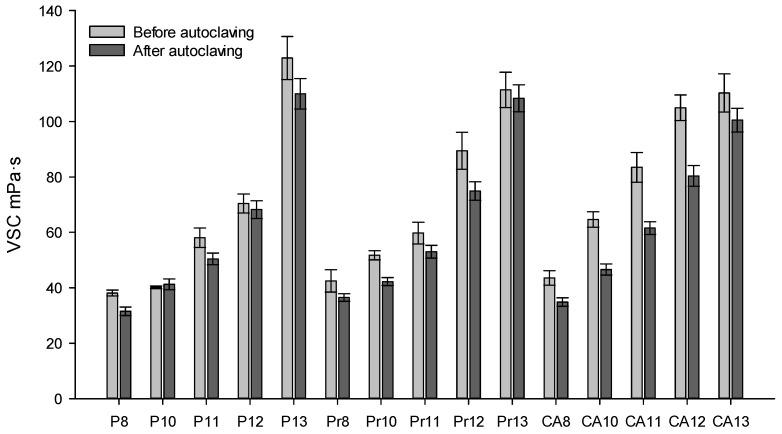
Viscosity (mPa·s) of experimental eye drops at room temperature (21 ± 1 °C) at the beginning of production and after sterilization using high-pressure steam.

Upon evaluating the viscosity of the experimental eye drops before sterilization, values ranged from 31.52 ± 1.55 to 110.00 ± 5.5 mPa·s (Figure 1). An increase in the concentration of the polymer Poloxamer 407 in the formulation was correlated with increased viscosity. A statistically significant difference in viscosity was observed between formulations containing 13% P407 compared to those with 8–12% P407 (*p* < 0.05). The results indicated that formulations with 8% P407 exhibited the lowest viscosity. Formulations with 10% P407 did not significantly differ in viscosity from those with 8% P407. Post-sterilization viscosity measurements of the modeled experimental in situ eye drops using high-pressure steam revealed viscosities ranging from 33.88 ± 1.32 to 108.73 ± 5.79 mPa·s. There was no statistically significant change in viscosity after sterilization with high-pressure steam (*p* < 0.05). An increase in viscosity was observed with higher concentrations of P407 in the formulations.

#### 2.3.2. Experimental Eye Drops’ pH Values and Sol-to-Gel Temperature

The pH of the modeled in situ eye drops ranged from 6.5 ± 0.3 to 7.0 ± 0.3, meeting the requirements for ocular solutions (Table 4). The *sol-to-gel* transition temperature of these in situ eye drops was evaluated, a crucial factor in assessing the quality and applicability of the formulations. Rheological analysis and assessment of the gelation point post-sterilization revealed that the P12, P13, Pr12, and Ca13 formulations exhibited in situ gel properties with temperatures close to the ocular surface, ranging from 31.9 ± 0.5 °C to 33.9 ± 0.2 °C. Formulations containing 11% Poloxamer 407 demonstrated a higher gelation temperature, from 36.4 ± 0.3 °C to 38.4 ± 0.3 °C. Remaining formulations showed gelation temperatures higher than 40 °C, in comparison to in situ gels with 11–13% P407 content.

### 2.4. In Vitro Release Test

**Figure 2:**
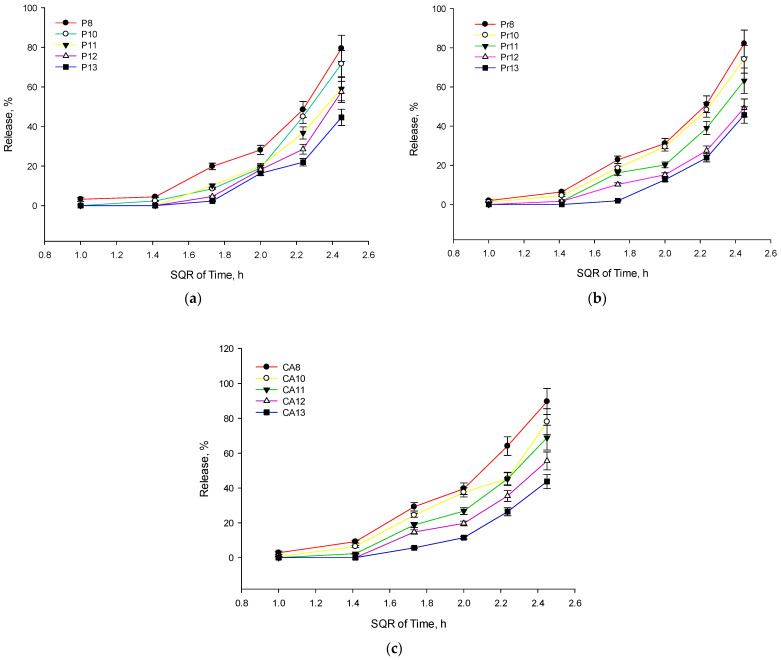
Higuchi equation for release kinetic (square root of time) of total phenolic compounds and *p*-coumaric acid from experimental eye drop formulations, mean ± SD, n = 3. Graph (**a**) shows release kinetics of formulations with balsam poplar bud extracts, graph (**b**)—propolis extracts, and graph (**c**)—with *p*-coumaric acid solutions.

The drug release increased with time for all formulations, as expected. The increase was not linear but appeared to follow a curve that might be characteristic of a diffusion-controlled process in a semi-solid matrix, which could fit the Higuchi release model. Evaluating the released total phenolic compound content, it was determined that formulations containing 8% P407 released the highest amount of active compounds (Figure 2). Formulations with 8% and 10% P407 released significantly more active compounds compared to those with 11–13% P407 (*p* < 0.05). This suggests that the drug is released more quickly from this formulation, which could indicate a less viscous matrix, facilitating drug diffusion. Formulations P11, P12, and P13 showed a more gradual release of the phenolic compounds or *p*-coumaric acid. The increase in drug release was less steep compared to formulations with 8 and 10% P407, which may imply a more controlled release rate. The difference in release rates among these formulations could have been due to varying matrix densities that slow down diffusion.

### 2.5. Experimental Formulations’ Stability after 90 Days

#### 2.5.1. Experimental Eye Drops’ Viscosity after 90 Days

**Figure 3:**
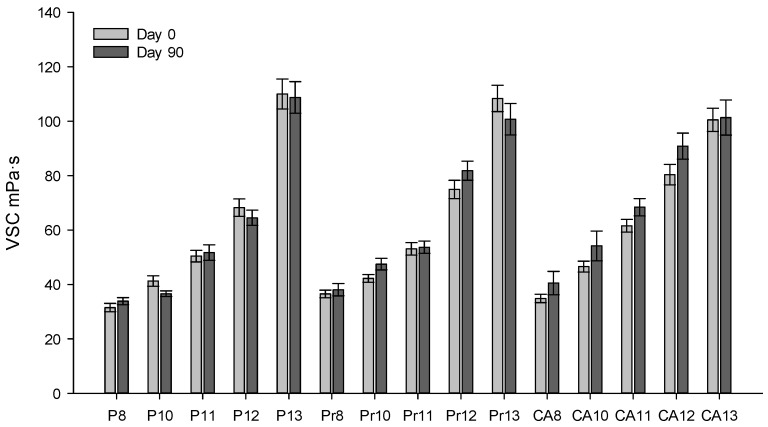
Viscosity (mPa·s) of experimental eye drops at room temperature (21 ± 1 °C) on production day 0 and 90 days post-production.

All experimental formulations were stored in a stability cabinet at 25 ± 2 °C with a relative humidity of 75 ± 5% for 90 days (Figure 3). After this period, the viscosity of the modeled in situ gels was evaluated. The study results indicated that the viscosity of the formulations did not change significantly. The formulations remained stable post-sterilization for 90 days.

#### 2.5.2. Experimental Eye Drops’ pH Values and Sol-to-Gel Temperature after 90 Days

**Figure 4:**
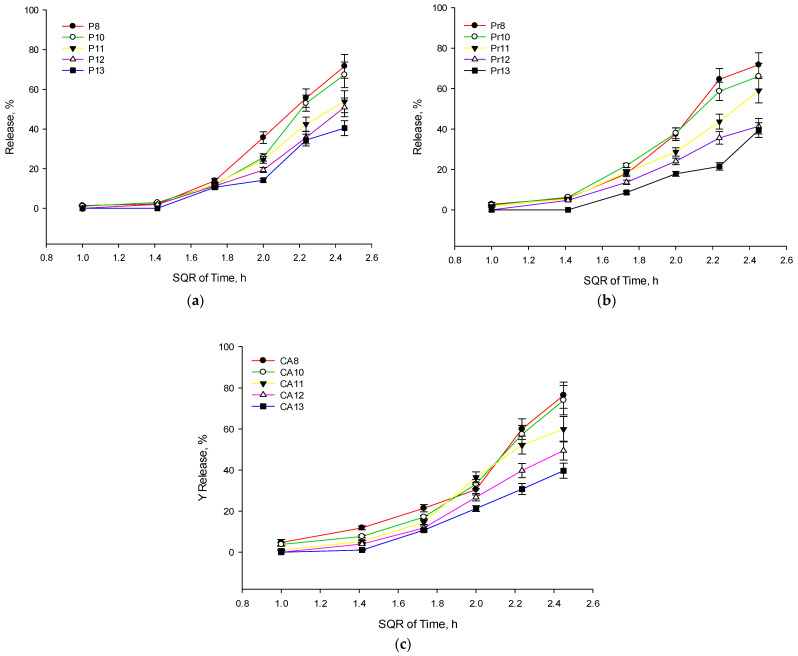
Higuchi equation for release kinetic (square root of time) of total phenolic compounds and *p*-coumaric acid from experimental eye drop formulations after 90 days post-production, mean ± SD, n = 3. Graph (**a**) shows release kinetics of formulations with balsam poplar bud extracts, graph (**b**)—propolis extracts, and graph (**c**)—with *p*-coumaric acid solutions.

To assess the stability of the formulations, other physicochemical parameters—pH value and sol-to-gel temperature—were evaluated (Table 5). After sterilization and 90 days in a stability cabinet (25 ± 2 °C, relative humidity 75 ± 5%), pH values ranged from 6.4 ± 0.0 to 7.1 ± 0.2. There was no significant change in the pH of the formulations. The gelation temperature remained stable after 90 days in the stability cabinet. Formulations containing 12–13% P407 exhibited a *sol-to-gel* transition temperature close to the ocular surface temperature. Upon evaluating the ability of the experimental formulations to release active compounds after 90 days in the stability cabinet, no significant change was observed in the release kinetics and the amount of active compounds (Figure 4).

## 3. Discussion

Ethanol-based propolis and plant extracts are commonly produced using organic solvents, but the use of such extracts orally or topically is not always ideal [22]. Our chemical composition studies show that aqueous solutions and aqueous solutions with polyethylene glycol 400 (PEG400) co-solvent addition are suitable for the extraction of propolis and poplar bud raw materials. The HPLC study results confirm that water-based solvent systems for propolis and poplar bud extraction are a good alternative to ethanol-based solvents [21]. The extracts were dominated by *p*-coumaric acid, a primary phenolic acid in Lithuanian propolis [19,23]. Based on chemical composition results, poplar bud extract appears to be a stronger source of the flavonoids pinocembrin, pinobanksin, and galangin compared to propolis. All identified active ingredients contribute to the biological effects of the extracts. Galangin exhibits anticancer, antimutagenic, antioxidant, bactericidal, and antifibrotic properties [24,25,26]. Pinocembrin, pinobanksin, and apigenin are known as strong antioxidants [27,28,29,30]. Vanillin, vanillic acid, and ferulic acid were identified in the propolis extract. Ferulic acid’s UV absorption catalyzes stable phenoxyl radical formation, enhancing its ability to terminate free radical chain reactions [31]. Vanillin showed stronger activity than ascorbic acid and Trolox in the ABTS [32]. Salicin, identified in poplar bud extract, is a strong antioxidant agent with in vivo anticancer activity [33]. The tested extracts exhibited similar SPF values, although the distribution of active compounds in propolis and poplar bud extracts was different, possibly due to the complex action of the compounds. Studies show that pre-treatment with *p*-coumaric acid resulted in substantially reduced cell death in human epidermal melanocytes exposed to UV compared to both untreated controls and cells treated post-exposure [34]. Additionally, *p*-coumaric acid has been found to mitigate oxidative damage in the eyes from UV light in both laboratory and animal studies [14,35]. Chemical composition analysis showed that both extracts are natural antioxidant agents and can potentially be applied in eye drop modeling.

The incorporation of polymeric materials into eye drop compositions helps address poor permeability issues [36]. In experimental studies, polymers were chosen to increase the viscosity of eye drops and prolong the release of embedded extract components. Poloxamer-based eye drop production was associated with in situ gel formulation. In situ gels have gained increasing interest as ocular drug delivery systems due to improved bioavailability [37]. Poloxamer 407 was selected for its thermoreversible gelation properties, which are advantageous for creating in situ eye drops that transition to a gel state at ocular temperatures. However, Poloxamer 407 alone lacks sufficient mucoadhesive qualities, which are essential for ensuring prolonged retention on the eye surface. To mitigate this, we incorporated sodium carboxymethylcellulose (NaCMC), a polymer known for its excellent mucoadhesive characteristics [38]. The combination of Poloxamer 407 and NaCMC aimed to balance the formulation’s thermogelling ability with enhanced mucoadhesion, potentially increasing the residence time and the possibility to improve the bioavailability of the administered therapeutic agents [37]. Eye drop solutions are well tolerated when their pH matches tear fluid [39]. Neutral or slightly alkaline eye drops are better tolerated than those with acidic pH [40]. The pH value of the produced formulations met the requirements for eye drops, with the optimal pH value for ophthalmic preparations ranging from 5.50 to 7.50 [41]. Study results confirmed that polymer addition increases viscosity [36]. With increasing poloxamer concentration, eye drop viscosity was higher. In the development of in situ eye drop formulations utilizing Poloxamer 407 (P407), the sol-to-gel transition temperature plays a pivotal role, particularly due to the inherent temperature of the ocular surface. P407, a thermosensitive polymer, undergoes a reversible phase transition from a low-viscosity solution (sol) at room temperature to a semi-solid gel upon exposure to higher temperatures, such as those found at the ocular surface (around 34–37 °C) [42]. This property is important for ensuring the formulation’s efficacy and patient comfort upon application. In the quality assessment of eye drops, attention to their microbiological contamination is crucial. Eye drops must be sterile for safe use. Pathogen-contaminated eye drops can cause serious eye infections such as endophthalmitis and keratitis [43].

Autoclave sterilization is a successful method to ensure the cleanliness of eye drops [44]. The study results showed that the selected polymers are resistant to autoclaving conditions, with no significant changes in the pH value, viscosity, or gelation point of the formulations post-sterilization. This finding underscores the compatibility of these polymers with standard sterilization procedures, ensuring that the sterility of eye drops does not compromise their physical or chemical integrity.

The results confirmed the hypothesis that incorporating polymers into eye drops as viscosity modifiers is a correct approach. As the polymer content increased, the release of active compounds in all three groups of eye drops slowed down. The application of viscosity modifiers is technologically simple, suggesting its wide applicability in the preparation process of eye drops [41]. This strategy not only enhances the drug’s contact time with the ocular surface but also facilitates controlled release, contributing to the overall effectiveness of the therapy.

Eye drops stored in sealed, sterilized packages remained stable for 90 days at a stability cabinet temperature of 25 °C. Stability tests included measurements of viscosity, gelation point, and pH values. The results indicated that the tested preparations maintained stability, with no significant changes in physical properties. To ensure the safety and efficacy of the prepared eye drops, detailed stability studies are crucial [45]. Future stability studies should include assessments of microbiological contamination and analyze the impact of different packaging, storage, and usage conditions on their stability [43].

## 4. Conclusions

Propolis and poplar bud extracts are natural sources of *p*-coumaric acid and other phenolic compounds and offer a broad research field in the development of eye drops. The pH values and viscosity of all eye drops fall within the comfort range for the eye and are unlikely to cause discomfort. Poloxamer-based eye drops are potential candidates for new in situ gel compositions. Based on research results, the recommended P407 concentration for in situ eye drops can be from 11 to 12%. The physical property fluctuations of the eye drops were statistically insignificant over three months, indicating stability. The practical application of experimental in situ gels necessitates biological studies of the formulations.

## 5. Materials and Methods

### 5.1. Materials

All reagents, standards, and solvents were of analytical grade. Purified deionized water was obtained using a Milli-Q^®^ water purification system (Millipore, Burlington, MA, USA). Ethanol (96% rectified) was sourced from JSC “Stumbras” (Kaunas, Lithuania). Folin–Ciocalteu’s reagent (Merck, Darmstadt, Germany); acetonitrile; and reference standards including chlorogenic acid, apigenin, vanillin, vanillic acid, ferulic acid, *p*-coumaric acid, caffeic acid, cinnamic acid, pinobanksin, pinocembrin, galangin, and salicin were acquired from Sigma-Aldrich (Steinheim, Germany). Sodium carbonate was procured from Sigma-Aldrich (Saint-Quentin-Fallavier, France). Poloxamer 407 and sodium carboxymethylcellulose were also obtained from Sigma-Aldrich (St. Louis, MO, USA).

### 5.2. Plant Material Extraction

Dried balsam poplar buds were sourced from Jadvyga Balvočiūtė’s organic herb farm, and propolis from Brolių medus. The poplar buds were collected in March 2022 in Lithuania. For the extraction of raw materials (poplar buds and propolis), purified water was used as the solvent. The ratio of raw material (poplar buds and propolis) to extractant was 1:10. The extraction was conducted using ultrasonic extraction at a temperature of 40 °C for a duration of 60 min. The extracts were then filtered through ashless filter paper to remove solids. Aqueous extracts were purified using sterile filtration, which ensured the removal of microorganisms and fine particulates, resulting in a clear liquid extract. Poplar bud and propolis extracts were stored in a refrigerator at 5 ± 1 °C, shielded from light until further stages of the research.

### 5.3. Assessment of Total Phenolic Content

The total phenolic content in extracts of balsam poplar buds and propolis was determined using a modified version of Singleton et al.’s method [46]. This involved the application of the Folin–Ciocalteu reagent for phenolic compound quantification. In a 25 mL volumetric flask, the reaction mixture was prepared by adding 1 mL of the extract, 9 mL of purified water, and 1 mL of Folin–Ciocalteu reagent. The mixture was blended, and after a wait of 2–3 min, 1.5 mL of 7.5% Na_2_CO_3_ solution was incorporated. The flasks were then filled to the 25 mL mark with purified water. The samples underwent a 40 min incubation at room temperature (21 ± 1 °C) in an environment shielded from light. Absorbance readings were taken at 760 nm using an Agilent Technologies 8453 UV-Visible Spectrophotometer (Santa Clara, CA, USA). The phenolic content was expressed as mg of *p*-coumaric acid equivalents per gram of dry weight (mg CAE/g, DW).

### 5.4. HPLC Analysis

The phenolic compounds in plant material extracts and in situ gels were qualitatively and quantitatively assessed using High-Performance Liquid Chromatography (HPLC) [19]. The analysis employed a Waters 2695 chromatographic system integrated with a Waters 996 photodiode array detector, using an ACE 5C18 chromatographic column measuring 250 × 4.6 mm. Data processing was conducted using Empower 2 Chromatography Data Software. The HPLC eluents consisted of 0.1% trifluoroacetic acid and of 100% acetonitrile. The elution program was used as follows: from 5% to 15% B at 0–8 min, from 15% to 20% B at 8–30 min, from 20% to 40% B at 30–48 min, from 40% to 50% B at 48–58 min, from 50% to 50% B at 58–65 min, from 50% to 95% B at 65–66 min, from 95% to 95% B at 66–70 min, and from 95% to 5% B at 70–71 min. The column temperature was maintained at 25 °C, with an injection volume of 10 µL, a mobile phase flow rate of 1 mL/min, a total run time of 81 min, and a particle size of 5 µm. The analysis utilized standard compounds including salicin (R^2^ = 0.9999), *p*-coumaric acid (R^2^ = 0.9999), caffeic acid (R^2^ = 0.9999), chlorogenic acid (R^2^ = 0.9999), cinnamic acid (R^2^ = 0.9999), pinocembrin (R^2^ = 0.9998), pinobanksin (R^2^ = 0.9999), apigenin (R^2^ = 0.9998), vanillin (R^2^ = 0.9999), vanillic acid (R^2^ = 0.9999), ferulic acid (R^2^ = 0.9999), and galangin (R^2^ = 0.9999). Compounds present in the samples were identified by comparing retention times and UV absorption profiles with those of the analytical standards.

### 5.5. Gelified Eye Drop Formulation

The formulation of gelified eye drops was developed using Poloxamer 407 (Kolliphor® P407, Sigma-Aldrich (St. Louis, MO, USA)) as the primary gelling agent, sodium carboxymethylcellulose (NaCMC), purified water, balsam poplar bud extract, propolis extract, and a *p*-coumaric acid solution (0.6 mg/mL in 20% ethanol (*v*/*v*). The poloxamer base was prepared using a cold process, where a specific amount of poloxamer was mixed with purified water and allowed to stand for 24 h in a refrigerator at 5 ± 1 °C. The NaCMC was prepared separately, with a predetermined amount of the polymer dissolved in an appropriate volume of purified water. The mixtures were continuously stirred at room temperature using a magnetic stirrer until a homogeneous, uniform, and transparent mass was obtained. In situ gels were formed by combining the Poloxamer 407 base with the NaCMC gels using a magnetic stirrer until a uniform mass was achieved. Balsam poplar bud extract, propolis extract, or *p*-coumaric acid solution was then incorporated into the prepared formulations, and the gels were mixed until uniform using a magnetic stirrer. The formed in situ gels were stored in sealed plastic containers in a refrigerator at 5 ± 1 °C.

### 5.6. Sterilization of Formulations Using High-Pressure Steam

The sterilization of the developed gelified eye drops was carried out using high-pressure steam sterilization for 20 min at 15 psi and 121 °C. Post-sterilization, the physicochemical parameters of the gelified eye drops were assessed, including pH, viscosity, physical appearance, and the concentration of active compounds.

### 5.7. Physicochemical Properties of In Situ Gels

The pH of the formulated gelified eye drops was measured using a pH meter (Knick SE 104N electrode) at room temperature, calibrated with buffer solutions in the pH range of 4.0–7.0. The viscosity of the gelified eye drops was determined using a vibrational viscometer (Vibro Viscometer SV-10, A&D Company Ltd., Tokyo, Japan) at room temperature (21 ± 1 °C).

### 5.8. Sol-to-Gel Transition Temperature

The sol-to-gel transition temperature of the ophthalmological preparations was determined using a Physica MCR 301 rheometer (Anton Paar GmbH, Graz, Austria). Measurements were conducted in the 20–50 °C range, with an amplitude of 0.5%, angular frequency of 10 rad/s, and a heating rate of 2 °C per minute. The sol-to-gel transition point was identified where the storage modulus (G′) intersected with the loss modulus (G″), analyzed using Rheoplus software 2.6x (Anton Paar GmbH, Ostfildern, Germany). G′ indicates the material’s elasticity, while G″ denotes its viscosity [47].

### 5.9. In Vitro Release Test

The release of active compounds was evaluated using Franz diffusion cells with cellulose semi-permeable membranes (25 mm, Sigma Aldrich, St. Louis, MO, USA). Prior to the experiment, the cellulose semi-permeable membranes were hydrated in purified water for 12 h. Purified water was used as the receptor medium, with a volume of 10 mL. During the release study, a constant temperature of 34 ± 1 °C was maintained, and the medium was continuously stirred using a magnetic stirrer. Samples of 1 mL were collected hourly from the receptor medium, with the final sample taken after 6 h. The amount of released bioactive compounds was determined spectrophotometrically by assessing the total phenolic content (the methodology is described in Section 5.3).

### 5.10. Assessment of Extracts’ Sun Protection Factor (SPF)

The Sun Protection Factor (*SPF*) of the extracts was determined using spectrophotometric methods, based on a modified approach by Oliveira-Júnior et al. [48]. During the analysis, the extracts were diluted in 96% ethanol to achieve a concentration of 50 µg CAE/mL. The absorbance spectra of the extract samples were recorded in the range of 290–450 nm using a 1 cm quartz cuvette. Absorbance data were collected from 290 to 320 nm, at 5 nm intervals. As a blank, 96% ethanol was used. The results were expressed as the arithmetic mean and standard deviation of three measurements.

The spectrophotometric *SPF* values were calculated using the following equation provided by Mansur et al. (1986) [49]:

(1) $$SPF=\mathrm{CF}\;\times \sum_{290}^{320}EE\left(\lambda \right)\times I\left(\lambda \right)\times Abs\;(\lambda )$$

*EE* (*λ*)—erythemal effect spectrum; I (*λ*)—solar intensity spectrum; *Abs* (*λ*)—absorbance of extract; CF—correction factor (=10).

The constants for the product of erythemal effect and solar spectrum intensity were evaluated according to Sayre et al. and are presented in Table 6 [50].

### 5.11. Statistical Analysis

Results are expressed as the mean and standard deviation of three measurements. The one-way ANOVA test was employed to ascertain statistically significant differences among the compared datasets. Post hoc analysis was conducted using Tukey’s multiple comparison test, with significance established at *p* < 0.05. Data processing and graphical representation were carried out using SigmaPlot 13.0 (Systat Software, San Jose, CA, USA) and IBM SPSS Statistics 27 (SPSS Inc., Chicago, IL, USA).

## Figures and Tables

**Table 1:** Total phenolic content in aqueous extracts of balsam poplar buds and propolis. Data are presented as mean (mg/mL) and standard deviation (SD), n = 3. The table also includes the SPF values of balsam poplar buds extracts, propolis extracts, and *p*-coumaric acid solution.

	Total Phenolic Compounds CAE mg/g	SD	SPF Value
Propolis extract	76.63	5.39	12.7
Balsam Poplar bud extract	83.25	3.88	14.0
*p*-coumaric acid solution	-	-	25.5

**Table 2:** HPLC analysis of aqueous extracts of balsam poplar buds and propolis. Data are presented as mean (µg/mL) and standard deviation (SD), n = 3.

	Balsam Poplar Bud Extract µg/mL	SD	Propolis µg/mL	SD
Salicin	112.07	4.99		
Chlorogenic acid	5.60	0.21		
Apigenin	17.75	0.64	6.79	0.48
Caffeic acid	26.42	1.09	42.22	2.95
*p*-coumaric acid	532.78	31.68	486.02	25.16
Cinnamic acid	101.30	4.75		
Pinobanksin	44.30	1.75		
Pinocembrin	125.43	4.71	80.67	6.90
Galangin	137.18	6.82		
Vanilin			105.60	5.58
Vanillic acid			17.58	0.90
Ferulic acid			251.78	19.00

**Table 3:** Composition of experimental eye drops containing balsam poplar bud extracts, propolis, and 0.6 mg/mL *p*-coumaric acid solution.

	Poloxamer 407	NaCMC	Active Compound	Purified Water
Group I	8	0.75	Balsam poplar bud extract 7.5	add 100
	10			
	11			
	12			
	13			
Group II	8	0.75	Propolis extract 7.5	
	10			
	11			
	12			
	13			
Group III	8	0.75	*p*-coumaric acid solution 7.5	
	10			
	11			
	12			
	13			

P—poplar buds, Pr—propolis, CA—*p*-coumaric acid. The numbers 8–13 indicate P407 concentration. Concentrations are expressed as % (*w*/*w*).

**Table 4:** Viscosity and pH values of experimental eye drops at room temperature (21 ± 1 °C) after sterilization using high-pressure steam and sol-to-gel transition temperature, mean ± SD, n = 3.

	P8	P10	P11	P12	P13	Pr8	Pr10	Pr11	Pr12	Pr13	CA8	CA10	CA11	CA12	CA13
pH	6.5	6.6	6.7	6.8	6.7	6.5	6.6	6.7	6.6	6.6	6.7	6.9	7.0	6.8	6.9
SD	0.3	0.4	0.3	0.3	0.3	0.3	0.3	0.3	0.3	0.3	0.3	0.3	0.3	0.3	0.4
*Sol-to-gel* (°C)	>40	>40	36.8	33.9	31.9	>40	>40	38.4	33.1	30.8	>40	>40	36.4	32.1	30.1
SD	-	-	0.3	0.2	0.5	-	-	0.3	0.2	0.3	-	-	0.3	0.4	0.5

**Table 5:** Viscosity and pH values of experimental eye drops at room temperature (21 ± 1 °C) after sterilization using high-pressure steam and *sol-to-gel* transition temperature, 90 days post-production.

	P8	P10	P11	P12	P13	Pr8	Pr10	Pr11	Pr12	Pr13	CA8	CA10	CA11	CA12	CA13
pH	6.4	6.5	6.7	6.6	6.5	6.5	6.6	6.7	6.7	6.7	6.6	7.0	7.1	7.0	7.0
SD	0.0	0.1	0.1	0.1	0.1	0.1	0.2	0.2	0.3	0.2	0.2	0.2	0.2	0.1	0.1
*Sol-to-gel* (°C)	>40	>40	38.4	34.4	32.5	>40	>40	>40	33.9	31.9	>40	>40	37.8	34.9	32.1
SD	-	-	0.8	0.4	0.4	-	-	-	0.3	0.2	-	-	0.5	0.3	0.4

**Table 6:** Normalized (*EE* × I) values for the erythemal effect and solar spectrum intensity [46].

	Wavelength, λ	EE × I
1	290	0.015
2	295	0.0817
3	300	0.2874
4	305	0.3278
5	310	0.1864
6	315	0.0839
7	320	0.018
Total		1

## Data Availability

The data presented in this study are openly available in article.
